# The Evolving Role of Neoadjuvant Radiation Therapy in Pancreatic Adenocarcinoma

**DOI:** 10.3390/jcm13226800

**Published:** 2024-11-12

**Authors:** John Michael Bryant, Justyn Nakashima, Vaseem M. Khatri, Andrew J. Sinnamon, Jason W. Denbo, Pamela Hodul, Mokenge Malafa, Sarah Hoffe, Jessica M. Frakes

**Affiliations:** 1 Department of Radiation Oncology, H. Lee Moffitt Cancer Center & Research Institute, Tampa, FL 33612, USA; 2 Department of Gastrointestinal Oncology, H. Lee Moffitt Cancer Center & Research Institute, Tampa, FL 33612, USA

**Keywords:** neoadjuvant radiation therapy, pancreatic ductal adenocarcinoma (PDAC), borderline resectable pancreatic cancer (BRPC), locally advanced pancreatic cancer (LAPC), surgical resection, stereotactic body radiation therapy (SBRT), hypofractionated radiation therapy, chemoradiation therapy (CRT)

## Abstract

Background/Objectives: Pancreatic ductal adenocarcinoma (PDAC) is one of the deadliest cancers. Surgical resection is the most reliable chance for cure, but high rates of positive margins and local failure persist. Neoadjuvant therapies (NAT), including chemotherapy and radiation therapy (RT), are being explored to improve surgical outcomes, particularly in borderline resectable (BRPC) and locally advanced pancreatic cancer (LAPC). This review aims to summarize the current landscape and future directions for neoadjuvant RT (NART) in PDAC. Methods: The review includes a detailed analysis of past and ongoing clinical trials investigating various NART approaches in PDAC, with an emphasis on different RT techniques, fractionation schemes, and their integration into multimodal treatment strategies. Results: Early evidence suggests that NART can improve resection margins and local control. However, recent trials, including the Alliance A021501 and LAP-07 trials, have failed to demonstrate significant survival benefits with the addition of RT to NAT. Nevertheless, nuances in trial design and execution continue to keep the question of NART open. Newer approaches, such as stereotactic magnetic resonance-guided adaptive radiation therapy (SMART), show promise in improving local control and survival, but further phase 3 trials are needed. Conclusions: While NART has shown potential in improving local control in PDAC, its impact on overall survival remains unclear. Ongoing trials, particularly those utilizing advanced techniques like SMART, are critical in defining the role of RT in the neoadjuvant setting for PDAC. Collaboration across multidisciplinary teams is essential to optimize treatment strategies and trial outcomes.

## 1. Introduction

Pancreatic ductal adenocarcinoma (PDAC) is one of the deadliest cancers worldwide, with a 5-year relative survival rate of 12.8% [1]. Surgical resectability and likelihood of a negative resection margin have always held paramount importance for the prognosis in PDAC management, as surgery is the most reliable chance for a cure. Surgery on its own typically results in a 40–80% positive margin rate [2,3], and up to half of patients will experience a local failure [4,5]. The standard of care for resectable PDAC is surgical resection, followed by adjuvant chemotherapy (CHT) [6]. Adjuvant FOLFIRINOX significantly improves overall survival in patients with resected PDAC, with a median survival of 54 months [7]. However, only 15% of patients are resectable at presentation, and up to 20% of those patients are found to be unresectable at the time of surgery [8]. Additionally, many patients are unable to receive FOLFIRINOX after resection due to complications relating to the surgery itself [9]. To address these challenges, neoadjuvant therapies (NAT) have been explored to improve surgical resection outcomes and overall outcomes for PDAC.

NAT, as opposed to an upfront surgery approach, has demonstrated an improvement in overall survival in certain cases for upfront unresectable PDAC [10,11,12,13]. The intent of NAT in PDAC is to transform previously unresectable tumors into resectable types, increase the likelihood of achieving negative margins during resection, helping to ensure the completion of systemic therapy [9]. Additionally, given the high rates of distant failures after surgery for resectable PDAC, neoadjuvant systemic therapy may also be able to treat any occult metastatic disease. NAT also helps to biologically select which patients may benefit from a surgical resection. This effect of biological selection influencing outcomes is highlighted by a difference in overall survival of approximately 21 months when patients are enrolled either before or after surgery, despite receiving similar adjuvant therapies [7,14]. Finally, patients are more likely to exhibit a better performance status before surgery; therefore, NAT can avoid the possibility of postoperative complications or delayed recovery precluding patients from receiving the desired full course of planned therapies [13,14,15,16,17].

In addition to systemic therapies, radiation therapy (RT) has been explored in the neoadjuvant setting for PDAC [9]. RT works by delivering ionizing radiation that induces DNA damage in tumor cells, primarily through double-strand breaks, leading to cell death, primarily through mitotic catastrophe. Fractionating RT—delivering radiation in multiple smaller doses—takes advantage of the cancer cells’ reduced capacity to repair DNA damage while allowing normal cells time to recover between doses. This approach enhances treatment tolerance, reduces side effects, and effectively targets tumor cells.

The addition of RT as part of an NAT paradigm in PDAC is controversial [9,18]. Unlike CHT, RT is a local modality and is believed to contribute to survival through its locoregional effects by downstaging the primary tumor, increasing the chances for a negative resection margin, and reducing the chances for a locoregional failure. In borderline resectable pancreatic cancer (BRPC), prospective evidence for neoadjuvant concurrent chemoradiation therapy (CRT) has demonstrated improvements in overall survival [13,19]. However, results from the more recent Alliance randomized trial of neoadjuvant sequential chemotherapy followed by hypofractionated radiation (25–40 Gy in 5 fractions) vs. neoadjuvant CHT did not demonstrate any benefits from the addition of RT [18]. Proponents of NART have brought up several limitations to the generalizability of the Alliance study’s results, including the lack of RT delivery conformity across the participating centers [9,20]. These differences in outcomes across prospective trials highlight the ongoing struggles to accurately define the role of NART in PDAC, the different approaches included within the NCCN guidelines, and the variation in treatment patterns across various cancer centers. To improve outcomes and help define the role of NART for patients with PDAC, several innovative trials are currently exploring various NART approaches. The currently recruiting phase 2 and/or phase 3 trials with an estimated enrollment of 50 or more patients exploring NART for PDAC registered on ClinicalTrials.gov are presented in Table 1. The currently recruiting phase 2 and/or phase 3 trials with an estimated enrollment of less than 50 patients, currently recruiting phase 1, and active but not-yet-recruiting trials are presented in Supplementary Table S1, Supplementary Table S2, and Supplementary Table S3, respectively.

With successful surgical resection as the only current reliable method to achieve a cure for patients with PDAC, the role of NAT is of paramount importance. However, the optimal neoadjuvant approach remains unclear. As opposed to CHT, RT presents more questions related to its role in the neoadjuvant setting for PDAC. Within this review, we summarize the current landscape and future directions for NART in PDAC.

## 2. Radiation Therapy in Pancreatic Adenocarcinoma

Early data suggested that combined modality therapy contributed to an improved overall survival in non-metastatic PDAC [21]. However, approximately one-third of patients were unable to receive adjuvant therapies due to delayed recovery after surgery [21]. As a result, NART was developed as a reasonable alternative and was commonly administered using a conventional fractionation scheme over the course of 5 to 6 weeks [22,23,24,25,26,27,28]. Due to high rates of rapid disease progression observed from the start of NART to surgery, a 2-week rapid-fractionation scheme, with an intraoperative boost, was developed to expedite NAT [29,30]. While this approach was developed to shorten overall treatment time, it also represents one of the earliest uses of hypofractionated RT (HFRT) in PDAC. Although the exact α/β of PDAC remains unclear [31], evidence suggests that a significant proportion of these tumors are more sensitive to a hypofractionated regimen [32,33]. The advent of intensity-modulated RT (IMRT) and image-guided RT (IGRT) during the early part of this century has further enhanced the feasibility of a hypofractionated and an ultra-hypofractionated approach, also called stereotactic body radiation therapy (SBRT).

Conventionally fractionated approaches have not been shown to significantly contribute to the overall survival for unresectable cases [34], which led to the exploration of both HFRT [35] and SBRT [36,37,38] strategies in PDAC. HFRT and SBRT are most often delivered sequentially as part of a multimodal approach to avoid excessive toxicities, although both have been explored with concurrent CHT [10,39]. In theory, the accelerated schedule and highly precise delivery of these approaches may be better suited as a part of a multimodal treatment approach for PDAC. A shortened overall treatment time minimizes the potential delay for surgery or more aggressive systemic therapy regimens, and a stereotactic approach allows for safer dose delivery. In fact, dose escalation using these approaches has been associated with improvements in survival [35,40]. However, the role of HFRT and SBRT in PDAC remains controversial [36,41,42,43,44,45].

While these approaches have generally demonstrated an improvement in local control, their association with survival has not been as consistent [35,36,41,42,43,44,45,46,47,48,49,50,51,52,53]. As previously stated, dose escalation is believed to be the key to improving survival for patients treated with RT and is thought to be especially important for those patients who never have surgery. Safe dose escalation has historically been challenging due to limited patient selection [35], given the proximity of nearby radiosensitive organs and the medical frailty of the patient population [54,55]. The advent of daily online MR-guided adaptive radiation therapy (MRgRT) and the combination MRI linear accelerator (MRL) has allowed for the safe delivery of ablative doses by overcoming many of the challenges associated with X-ray/CT-based SBRT [40,56,57]. Of note, CT-based daily adaptive therapy has recently demonstrated a favorable toxicity profile when treating patients with 40 Gy in five fractions; however, this still falls short of the typical 50 Gy in five fractions that is able to be safely delivered via an MRL [58].

The advantages of daily adaptive MRgRT using an MRL include improved soft-tissue visualization, direct gating of both the targets and nearby organs at risk, and real-time plan adaptation at each fraction [59]. Additionally, the dosimetric advantages of daily replanning also contribute to better overall target coverage and lower doses of OAR than what may be seen on the baseplan [59]. Given these advantages, stereotactic magnetic resonance-guided adaptive radiotherapy (SMART) has been primarily explored as a definitive treatment option for BRPC and LAPC [40,57,60,61,62,63]. Initial studies exploring A-SMART for BRPC and LAPC demonstrated limited toxicity and improved clinical outcomes [40,60,61,63]. These outcomes also appear to hold true for elderly and medically frail patients, i.e., those at the highest risk for treatment-related toxicities [57,63].

## 3. Upfront Resectable Pancreatic Adenocarcinoma

Resectability status serves as a key prognostic indicator for patients with PDAC. While institutions have historically defined resectability criteria individually, the NCCN has established standardized criteria to promote consistency in reporting across centers. The NCCN definitions for resectability status are presented in Table 2. A surgery first approach for patients with upfront resectable disease has long been considered a reasonable approach [6]. A surgery first approach typically results in a positive margin rate that ranges from 30 to 50% [64], although successful completion of adjuvant modified FOLFIRINOX is associated with a median overall survival of 54.4 months [7]. However, the study design that demonstrated that impressive overall survival rate with adjuvant modified FOLFIRINOX enrolled patients after surgery, thereby selected only the most medically fit patients exhibiting tumors with a less aggressive biology [9]. Therefore, the generalizability of this approach is limited, as is highlighted by SWOG 1505, which enrolled their patients prior to surgery and demonstrated that only approximately 70% of patients ultimately made it to surgery; therefore, there were no outcome benefits from biological selection alone [14]. Early enrollment resulted in a median overall survival rate of only 23.2 months [14]. This helps to explain that despite adjuvant trials demonstrating the most impressive survival outcomes, most NCCN member institutions actually prefer a neoadjuvant approach [6], which helps to biologically select for which patients would benefit the most from surgical resection [65].

Early evidence demonstrated that neoadjuvant CRT was feasible and safe, with a high negative margin resection rate and a major histopathological response [27,66,67]. These early data helped to establish the significant association between an R0 resection and improved survival [27,66,67]. The PREOPANC-1 provided some of the earliest prospective randomized evidence for the use of NART for upfront resectable PDAC. PREOPANC-1 was a multicenter phase 3 trial that explored concurrent CRT vs. upfront surgery in resectable and BRPC. In total, 246 patients were enrolled and randomized to either neoadjuvant concurrent gemcitabine-based CRT of 36 Gy in 15 fractions, followed by surgery and four cycles of adjuvant gemcitabine, or upfront surgery and six cycles of adjuvant gemcitabine for patients with resectable and BRPC. An improvement in median survival (15.7 vs. 14.3 months; *p* = 0.025) and a higher rate of R0 resection (72% vs. 43%; *p* < 0.001) was observed. However, in a subgroup analysis of resectable disease, there were no differences in survival rates. Given these results and the use of an outdated CHT regimen, most centers have not changed their approach to resectable PDAC. Therefore, both upfront surgery followed by CHT and a neoadjuvant paradigm that includes RT are considered to be acceptable treatment approaches [6].

## 4. Upfront Unresectable Localized Pancreatic Adenocarcinoma

The importance of surgical resection for patients with localized PDAC is difficult to overstate, as patients with upfront unresectable disease have similar survival outcomes compared to those for patients with upfront resectable PDAC, if they can obtain a successful surgical resection [68,69,70]. Understanding this nuance introduces an important factor when weighing potential NAT approaches, as many of these upfront unresectable patients will not make it to surgery. Although 50–60% of patients with BRPC [71] will eventually undergo a resection [18], this is not true for patients with locally advanced PDAC (LAPC [72]), who make up one-third of all newly diagnosed patients [73] and only exhibit a 15–20% surgical resection rate [10,74,75]. Therefore, the benefits of aggressive therapy must be carefully weighed against the risk of treatment-related toxicities that could influence surgical management in order to optimize outcomes for all patients with upfront localized unresectable PDAC.

It is important to note that the definitions for both BRPC and LAPC have been variable [76], and that resectability status is ultimately determined by the surgical oncologist. The important distinction between BRPC and upfront resectable PDAC is that these features portend a high risk of positive margins, increased surgical morbidity, and occult metastatic disease. Therefore, the role of NAT is to address these challenges by improving the chance for a successful complete resection and reducing the chance of distant disease progression. Given the increased complexity of these cases, the NCCN recommends that patients with upfront unresectable disease be treated or coordinated through a high-volume cancer center [6].

As for upfront resectable disease, systemic therapy represents the backbone of an NAT approach to the treatment of upfront unresectable disease [6], whereas the role of RT is more controversial [18]. After staging has been completed and if the patient displays a good performance status, then systemic therapy is often started and continued for a total of 6 months, with regular restaging performed. If there is evidence of response without metastatic disease and the surgical oncologist feels that a successful resection can be performed, then the patient is often taken to surgery at that time, with any remaining systemic therapy given in the adjuvant setting. Both the role and approach of RT in this setting is highly variable and often institutionally specific.

Early evidence demonstrated improved outcomes with NART in the treatment of upfront unresectable disease. Jang et al. performed one of the first prospective trials to evaluate the role of NAT in patients with BRPC. This was a multicenter randomized phase 2/3 trial that evaluated neoadjuvant gemcitabine-based CRT vs. adjuvant CRT of 54 Gy in 30 fractions. This study showed significant improvement in 2-year overall survival in the neoadjuvant CRT arm compared to the upfront surgery arm, with a median OS improvement of 21 vs. 12 months (*p* = 0.028). The R0 rate was also twice as high with neoadjuvant CRT (51.8% vs. 26.1%; *p* = 0.004) [13]. In addition to the specifications of Jang et al., the previously mentioned PREOPANC-1 trial also included patients with BRPC that presented similar improvements in R0 resection rates and improvements in overall survival [10].

To build upon the success of neoadjuvant CRT, a total NAT (TNT) approach was evaluated in a single-arm, phase 2, single-institution study by Murphy et al. to determine if preoperative therapy intensification can further improve the R0 resection rate [77]. Within this treatment paradigm, patients received FOLFIRINOX for four cycles, and if there was no progression noted on the restaging scans, an additional four cycles were administered. This was then followed by CRT of 25 Gy in 5 fractions with protons or 30 Gy in 10 fractions with photons with concurrent capecitabine. Long-course radiation was prescribed to 50.4 Gy in 28 fractions with concurrent capecitabine. This treatment intensification approach yielded an R0 resection rate of 67% [77]. An Italian TNT single-arm prospective trial by Maggino et al. was performed to evaluate the real-world outcomes of primary CHT in patients with upfront unresectable localized PDAC. RT was an independent risk factor (hazard ratio [HR] 0.700; 95% CI 0.563–0.872; *p* = 0.001) for disease-specific survival (DSS), along with completion of CHT (HR 0.390, 95% CI 0.319–0.477, *p* < 0.001) and surgical resection (HR 0.178, 95% CI 0.125–0.253, *p* < 0.001) [78]. Although RT was independently associated with improvements in DSS, both CHT and surgery demonstrated stronger associations [78].

Despite the favorable outcomes associated with RT seen in single-arm neoadjuvant studies, early randomized studies raised questions regarding the added benefit of RT to CHT for patients with unresectable PDAC [79,80]. In the French prospective GERCOR studies in 2007, Huguet et al. demonstrated that CRT after initial CHT prolonged the median overall survival rate to 15 months, compared to 11.7 months with CHT alone [81]. This became a common treatment sequence for patients with unresectable disease. However, more recent trials have presented contradictory results. In the French phase 3 FFCD-SFRO trial, a treatment with a CRT of 60 Gy in 30 fractions with concurrent 5-FU and cisplatin, followed by maintenance gemcitabine, was compared to that using gemcitabine alone. This trial resulted in superior 1-year overall survival in the CHT-alone arm [34]. On the other hand, the ECOG trial, which compared a CRT of 50 Gy in 28 fractions with concurrent gemcitabine, followed by maintenance gemcitabine, to treatment with gemcitabine alone, found that CRT significantly prolonged overall survival (11.1 vs. 9.2 months; *p* = 0.017) [82]. In the German CONKO-007 trial, 525 patients underwent induction CHT and were then randomized to either CHT alone or CRT with gemcitabine. R0 resection rates were significantly higher in the CRT arm (69% vs. 50%; *p* = 0.04). The pathologic complete response rate was also significantly higher in the CRT arm (18% vs. 2%; *p* = 0.004) [83].

Alliance A021101 was a multi-institutional phase 1 feasibility trial for patients with BRPC to evaluate accrual, tolerability, and treatment completion [84]. Patients underwent 4 months of mFOLFIRINOX and CRT to 50.4 Gy, followed by resection. The results showed that 68% of patients made it to surgery. The regimen was tolerable, although there was a G3+ toxicity rate of 64% [84]. The success of the feasibility trial led to the randomized phase 2 Alliance A021501 trial, which evaluated the addition of neoadjuvant sequential CRT to CHT alone [41]. However, as opposed to the protocol in the A021101 trial, conventional RT was not utilized, instead being replaced with a hypofractionated (25 Gy in 5 fractions) or SBRT (33-40 Gy in 5 fractions) regimen. Patients received either eight cycles of modified FOLFIRINOX or seven cycles of modified FOLFIRINOX, followed by either 33–40 Gy or 25 Gy in five fractions. Patients without evidence of disease progression proceeded to a pancreatectomy, followed by four cycles of adjuvant modified FOLFOX. The CRT arm was closed early due to a lack of benefits, with a median OS of 17.1 months, with 33% of patients displaying an R0 resection. This is compared to the results for neoadjuvant CHT alone, which exhibited a median OS of 29.8 months and an R0 resection rate of 57% [18]. The addition of CRT leading to a decrease in the R0 resection rate is a striking outcome, given the plethora of prior evidence suggesting a direct benefit of RT for successful resection. The authors of this study were unable to explain this finding, but predefined protocol criteria required the CRT arm’s early closure [18]. The PREOPANC-2 trial was another phase 3 randomized trial that compared neoadjuvant FOLFIRINOX to gemcitabine-based neoadjuvant CRT and adjuvant gemcitabine CHT for BRPC and upfront resectable PDAC [85]. This study did not demonstrate a statistically significant difference between the neoadjuvant gemcitabine-based CRT with adjuvant gemcitabine and the neoadjuvant FOLFIRINOX for either overall survival (21.9 vs. 21.3 months; *p* = 0.28) or resection rate (77% vs. 75%; *p* = 0.69) [86].

The results of the four-arm multicenter ESPAC-5f phase 2 trial, although not designed to directly compare neoadjuvant CHT to RT, also raised questions regarding the value of NART. In this study, 90 patients were randomized across four arms to different NAT modalities for patients with BRPC. These arms consisted of (1) upfront surgery, (2) two cycles of gemcitabine/capecitabine, (3) four cycles of FOLFIRINOX, and (4) CR at 50.4 Gy in 1.8 Gy per fraction, with concurrent capecitabine. In general, NAT demonstrated a strong overall survival advantage compared to upfront surgery (1 year OS: 39% vs. 77%; *p* < 0.001). However, the CRT arm fared worse than the two CHT arms, with a 1-year OS of 60% compared to 78% for gemcitabine plus capecitabine and 84% for FOLFIRINOX [12]. It should be noted that recruitment was limited, and each arm was comprised of a relatively small cohort of patients, which raises questions regarding the applicability of the conclusions drawn from the study [12].

The LAP-07 trial, which evaluated the addition of CRT to induction CHT in LAPC, also failed to demonstrate a survival benefit. LAP-07 was an international phase 3 randomized trial designed to test both erlotinib and CRT. The initial randomization was designed to evaluate erlotinib, and the second randomization evaluated CRT, if there was no evidence of disease progression after 4 months of induction systemic therapy. The CRT consisted of 54 Gy in 30 fractions, with concurrent capecitabine. CRT did not demonstrate a benefit in OS (15.2 months vs. 16.5 months with CHT alone; *p* = 0.83). However, CRT was associated with an overall decreased rate of local progression (32% vs. 46%; *p* = 0.03), without any significant increases in grade 3+ toxicity (except for nausea).

Despite the lack of benefits seen in recent prospective studies for the addition of RT to NAT in BRPC, uncertainties related to RT volume coverage [48,87] and surgical expertise after RT [9] keep the question of NART open. One of the biggest conundrums within the published data is the drastic difference in R0 rates between prospective trials and single-institution reports from high volume centers [62,88,89]. One of the arguments is that a lack of experience with ablative stereotactic RT and/or surgical resection of high-risk tumors in some participating centers included in the studies has contributed to the inferior outcomes compared to those reported by high volume centers with more experience [62,88,89]. With respect to the recent Alliance A021501 trial, a post hoc analysis revealed that 59% of radiation treatment plans revealed protocol deviations, with 28.2% being considered major deviations [20].

## 5. Future Directions

Future studies (Supplementary Table S3) are continuing to assess the role of NART in PDAC, including PANDAS-PRODIGE 44 (NCT02676349), which is a phase 2 trial evaluating patients with BRPC randomized to neoadjuvant mFOLFIRINOX, with or without CRT. Compared to A021501, this trial will be using conventional radiotherapy to 50.4 Gy with capecitabine. Additionally, the Nano-SMART trial (NCT04789486) at the Dana–Farber Cancer Institute is exploring the integration of gadolinium-based nanoparticles with SMART for LAPC. The PACER trial (NCT03716531) at Massachusetts General Hospital is evaluating electron beam intraoperative radiation therapy following CRT in patients with vascular involvement, and the SHAPER trial (NCT04106856) at the University of Utah is testing a combination of losartan and hypofractionated RT following induction CHT for patients with upfront unresectable disease. These innovative trials highlight the ongoing efforts to refine neoadjuvant therapy protocols and improve outcomes for PDAC patients through advanced radiation techniques and combination therapies.

One of the more promising areas of technological advancement within radiation oncology is the advent of the MRL and SMART [56]. An early multi-center retrospective study by Rudra et al. demonstrated that the safe dose-escalation made possible with MRgRT was associated with improvements in survival [40]. Upon univariate analysis, a biologically effective dose (α/β = 10; BED_10_) of a >70 Gy dose (HR 0.44; 95% CI 0.21–0.94; *p* = 0.03) was associated with improvements in OS. Additional early retrospective evidence continued to point towards improvements in both disease control and survival associated with ablative SMART for patients with PDAC, with a favorable safety profile [40,57,60,62,63,90], even in the elderly and medically frail patient populations [57]. SMART is able to safely and quickly deliver an ablative dose of radiation equal to a BED_10_ of 100 Gy (i.e., 50 Gy in five fractions delivered daily) that does not appear to impact the success of subsequent surgery [75,90]. This ability makes it a particularly attractive option for NART for upfront unresectable PDAC patients.

To date, there have been two phase 2 single-arm trials evaluating SMART in this patient population. The results of the multicenter SMART for Locally Advanced Pancreatic Cancer (NCT03621644) study, which consisted of 136 patients with either BRPC or LAPC, demonstrated a median overall survival of 22.8 months and a 1-year overall survival rate of 94% [90,91]. The Danish phase 2 trial, which consisted of 28 patients with LAPC, demonstrated similar results, with a median OS of 20.8 months from diagnosis [75]. Importantly, these prospective trials closely reflect the outcomes reported in the SMART retrospective data series [40,57,60,62,63,90]. Given these exciting early reports, there is a growing effort to explore ablative dose radiation in a randomized prospective manner after neoadjuvant CHT for upfront unresectable cases.

These innovative trials and the promising advancements in radiation oncology underscore a rapidly evolving landscape for neoadjuvant therapy in PDAC. As the field moves towards integrating more sophisticated technologies and targeted therapies, such as SMART and MR-guided RT, the therapeutic potential for otherwise challenging PDAC cases continues to expand. In addition to these promising developments, the characterization of PDAC’s resistance mechanisms, including the role of NRF2 and the KRAS-driven microenvironment, is helping to shape the future of combination strategies. Addressing these intrinsic resistance pathways may ultimately broaden the applicability and efficacy of NART. By pairing radiation with recent novel targeted therapies, the approach to PDAC could be fundamentally transformed, pushing us closer to more effective, tailored therapeutic strategies.

The activation of KRAS mutations, notably KRAS G12D and G12V, drives tumorigenesis in PDAC in >90% of tumors, thus making it a particularly promising target for novel therapies. However, the clinical application of this therapy faces challenges, including the complex tumor microenvironment, a lack of effective biomarkers to identify responsive tumors, and induced resistance to KRAS inhibitors [92,93]. Combination chemotherapy using gemcitabine/(n)ab-paclitaxel (GnP) with MRTX1133, a KRAS G12D inhibitor, showed a significant reduction in relapse and longer tumor growth inhibition in both primary and metastatic settings [92]. MTRX1133 is currently being evaluated in a phase I/II clinical trial for advanced solid tumors (NCT05737706). More recent studies have shown the synergistic effects of combining radiotherapy and small molecule inhibitors, specifically through the use of KRAS inhibitors [94,95]. Through DNA damage and altering the tumor microenvironment, these combination therapies have the potential to enhance the sensitivity of pancreatic tumor cells to targeted treatments. As research progresses, with growing evidence of the safety of combining RT and targeted therapy [96], treatments combining KRAS inhibitors with other therapeutic modalities, such as radiotherapy, immunotherapy, and targeted treatments, may enhance their effectiveness and provide a new promising therapeutic strategy for PDAC.

Studies have identified that NF-E2-related factor 2 (NRF2), a transcription factor, binds to antioxidant DNA response element (ARE) and reduces the burden of reactive oxygen species. NRF2 has been shown to upregulate following single and multiple fractions of radiation in a dose-dependent fashion, which may be a key mechanism of PDAC’s radioresistance [97]. A prevalent mutation in PDAC is KRAS G12D, which has also been shown to upregulate NRF2 expression and induce gemcitabine resistance, requiring higher doses for equivalent cell kill. This has translated to a significant difference in the survival rates of PDAC patients, depending on NRF2 expression in the tumor samples [98]. The loss of NRF2 led to increased radiosensitivity, and inhibition of glutamine metabolism, which was seen to be increased by NRF2 expression, led to increased gemcitabine sensitivity. Therefore, combination strategies addressing the known therapeutic resistance mechanisms may enhance the utility of neoadjuvant radiation therapy for PDAC.

## 6. Conclusions

Managing PDAC with RT, especially within an NAT paradigm, remains complex and evolutionary. Although surgical resection is the most reliable curative option, high local and distant failure rates highlight the need for additional therapies. While systemic therapy has clear benefits, the role of RT remains uncertain. As systemic efficacy improves, locoregional control will likely become more crucial for disease and survival outcomes. Advanced techniques like SMART and a promising combinational therapy with novel targeted agents offer promising avenues to explore.

## Figures and Tables

**Table 1:** Actively recruiting phase 2 and/or phase 3 trials with estimated enrollment of ≥50 patients incorporating neoadjuvant radiation therapy for pancreatic adenocarcinoma registered on ClinicalTrials.gov.

Study Title	Sponsor	Resectability Status	Estimated Enrollment	ClinicalTrials.gov Identifier
Preoperative Fractionated Radiation Therapy Versus Stereotactic Body Radiation Therapy for Resectable or Borderline Resectable, or Locally Advanced Type A Pancreatic Adenocarcinoma	Medical College of Wisconsin	Resectable, Borderline Resectable, or Locally Advanced	102	NCT03704662
Proton Radiation for Unresectable, Borderline Resectable, or Medically Inoperable Carcinoma of the Pancreas	Proton Collaborative Group	Borderline Resectable or Locally Advanced	60	NCT02598349
Concurrent Chemoradiotherapy Combined With Immunotherapy in Patients With Potentially Resectable Pancreatic Cancer	The Affiliated Nanjing Drum Tower Hospital of Nanjing University Medical School	Potentially Resectable	62	NCT05634564
Nano-SMART: An Adaptive Phase I-II Trial of AGuIX Gadolinium-based Nanoparticles With Stereotactic Magnetic Resonance-guided Adaptive Radiation Therapy for Centrally Located Lung Tumors and Locally Advanced Unresectable Pancreatic Ductal Adenocarcinoma	Dana–Farber Cancer Institute	Locally Advanced	100	NCT04789486
Preoperative Treatment With mFOLFIRINOX (or Gem-Nab-P) +/− Isotoxic High-dose Stereotactic Body Radiation Therapy (iHD-SBRT) for Borderline Resectable Pancreatic Adenocarcinoma: a Randomised Phase II Study (STEREOPAC)	Erasme University Hospital	Borderline Resectable	256	NCT05083247
Initial Feasibility Study to Evaluate the Safety and Efficacy of the Permanently Implantable LDR CivaSheet^®^ in Combination With External Beam Radiation in the Treatment of Pancreatic Cancer	CivaTech Oncology	Borderline Resectable or Locally Advanced	80	NCT02843945
Comparisons of Different Neoadjuvant Chemotherapy Regimens With or Without Stereotactic Body Radiation Therapy for Borderline Resectable Pancreatic Cancer: Study Protocol of a Prospective, Randomized Phase II Trial	Changhai Hospital	Borderline Resectable	150	NCT03777462
A Phase II Study on the Safety and Efficacy of PD-1 Combined With Nab-paclitaxel/Gemcitabine and PULSAR in the Treatment of Locally Advanced Unresectable Pancreatic Cancer and Local Recurrence After Surgery	Fudan University	Locally Advanced	81	NCT06359275
Phase II Study to Assess the Interest of a Sequential Treatment With Gemcitabine/Nab-paclitaxel (GEMBRAX) and Then FOLFIRINOX Followed by Stereotactic Magnetic Resonance-guided Adaptive Radiotherapy in Patients With Locally Advanced Pancreatic Cancer	Institut du Cancer de Montpellier—Val d’Aurelle	Locally Advanced	103	NCT04570943
A Phase 2, Open-Label, Multicenter, Randomized Study Evaluating Neoadjuvant Therapy Targeting the Adenosine Immunosuppressive Pathway in Combination With Immune Checkpoint Blockade and Radiation Therapy in Patients With Advanced PANCreatic Ductal Adenocarcinoma Who Are Candidates for Surgical Resection	Gulam Manji (Columbia University)	Resectable	60	NCT06048484
NeoOPTIMIZE: An Open-Label, Phase II Trial to Assess the Efficacy of Adaptive Switching of FOLFIRINOX or Gemcitabine/Nab-Paclitaxel as a Neoadjuvant Strategy for Patients With Resectable and Borderline Resectable/Locally Advanced Unresectable Pancreatic Cancer	OHSU Knight Cancer Institute	Resectable, Borderline Resectable, or Locally Advanced	60	NCT04539808
Total Neoadjuvant NALIRIFOX Plus Ablative Dose Radiation Therapy for Borderline Resectable and Locally Advanced Pancreatic Adenocarcinoma	Memorial Sloan Kettering Cancer Center	Borderline Resectable or Locally Advanced	60	NCT05851924
Sequential Neoadjuvant Chemotherapy for Borderline Resectable and Locally Advanced Pancreatic Adenocarcinoma	Wake Forest University Health Sciences	Borderline Resectable or Locally Advanced	64	NCT05825066
Stereotactic Adaptive Radiation Therapy of Borderline Resectable Pancreatic Cancer an Individualized Approach (ARTIA-Pancreas)	Varian	Borderline Resectable or Locally Advanced	134	NCT05764720

**Table 2:** NCCN Pancreatic Adenocarcinoma Resectability Criteria.

Resectability Status	Arterial Involvement	Venous Involvement
Resectable	No arterial contact	No SMV or PV contact ≤180° contact without vein contour irregularity
Borderline Resectable	≤180° contact with SMA or CA Contact with CHA without extension to CA or CHA bifurcation Contact with variant artery anatomy	Reconstructable SMV/PV: >180° contact with SMV or PV ≤180° contact with contour irregularity of vein with suitable vessel proximally and distally Contact with inferior vena cava
Locally Advanced	>180° contact with SMA or CA Contact with CA and aortic involvement (body/tail)	Unreconstructable SMV/PV

Abbreviations: SMA: superior mesenteric artery; CA: celiac artery; CHA: common hepatic artery; SMV: superior mesenteric vein; PV: portal vein.

## Data Availability

Not applicable.

## Supplementary Materials

The following supporting information can be downloaded at: https://www.mdpi.com/article/10.3390/jcm13226800/s1, Table S1: Actively recruiting phase 2 and/or phase 3 trials with estimated enrollment of <50 patients incorporating neoadjuvant radiation therapy for pancreatic adenocarcinoma registered on ClinicalTrials.gov; Table S2: Actively recruiting phase 1 trials incorporating neoadjuvant radiation therapy for pancreatic adenocarcinoma registered on ClinicalTrials.gov; Table S3: Active but not-yet-recruiting trials incorporating neoadjuvant radiation therapy for pancreatic adenocarcinoma registered on ClinicalTrials.gov.
