# Supplementary material for: The Evolving Role of Neoadjuvant Radiation Therapy in Pancreatic Adenocarcinoma
Source: J Clin Med. 2024 Nov 12;13(22):6800. doi: 10.3390/jcm13226800 (PMC11594810; doi:10.3390/jcm13226800)
Supplement: Supplementary file 1 [file jcm-13-06800-s001.zip › jcm-3282796-supplementary.pdf]

Supplementary Table S1. Actively recruiting phase 2 and/or phase 3 trials with estimated enrollment of < 50 patients incorporating neoadjuvant radiation therapy for pancreatic adenocarcinoma registered on ClinicalTrials.gov.

Study Title	Sponsor	Resectability Status	Estimated Enrollment	ClinicalTrials.gov Identifier
Adaptive Approach to Neoadjuvant Therapy to Maximize Resection Rates for Pancreatic Adenocarcinoma	University of Cincinnati	Resectable or Borderline Resectable	32	NCT04594772
Stereotactic Body Radiotherapy and Focal Adhesion Kinase Inhibitor in Advanced Pancreas Adenocarcinoma	Washington University School of Medicine	Borderline Resectable or Locally Advanced	42	NCT04331041
GEM+Nab-Paclitaxel Plus Losartan Followed by Stereotactic Radiotherapy for Locally Advanced Pancreatic Cancer (OVERPASS)	Istituto Scientifico Romagnolo per lo Studio e la cura dei Tumori	Locally Advanced	34	NCT05861336
Radiotherapy for Locally Advanced Pancreatic Carcinomas (Phase II Trial) (LAPC)	EBG MedAustron GmbH	Locally Advanced	30	NCT05191940
Preoperative Stereotactic Body Radiation Therapy in Patients With Resectable Pancreatic Cancer	Yonsei University	Resectable	25	NCT05679583
To Explore the Efficacy of Hypofractionated Radiotherapy Followed by AG Regimen Chemotherapy Plus Camrelizumab Immunotherapy as Neoadjuvant Therapy for Locally Advanced Pancreatic Cancer	Hebei Medical University Fourth Hospital	Locally Advanced	20	NCT06435260
Split-course SBRT for Borderline Resectable and Locally Advanced Pancreatic Cancer	Fujian Medical University Union Hospital	Borderline Resectable or Locally Advanced	27	NCT04289792
Testing the Combination of Two Approved Chemotherapy Drugs and Radiation Prior to Surgery in Localized Pancreatic Cancer	Loma Linda University	Resectable	30	NCT03492671
Stereotactic Body Radiotherapy [SBRT] for High Risk Localised Pancreatic Cancer: a Phase II Study of the Department of Radiation Oncology Royal North Shore Hospital (Span-C - SBRT for Pancreatic Cancer)	Royal North Shore Hospital	Borderline Resectable or Locally Advanced	40	NCT03505229

DP-IMRT Pancreas: A Non-randomised Phase I/II Study of Dose-escalated Hypofractionated Dose-Painted Intensity Modulated Radiotherapy (DPIMRT) in Resectable/Borderline Resectable Pancreatic Adenocarcinoma	Cancer Trials Ireland	Resectable or Borderline Resectable	49	NCT06024824
A Randomized Phase II Study of the Efficacy and Safety of Hypofractionated Stereotactic Radiotherapy and 5FU or Capecitabine With and Without Zometa in Patients With Locally Advanced Pancreatic Adenocarcinoma	University of Nebraska	Resectable, Borderline Resectable, or Locally Advanced	44	NCT03073785
A Phase I/II Trial of Combination Immunotherapy With Nivolumab and a CCR2/CCR5 Dual Antagonist (BMS-813160) With or Without GVAX Following Chemotherapy and Radiotherapy for Locally Advanced Pancreatic Ductal Adenocarcinomas (PDACs).	Sidney Kimmel Comprehensive Cancer Center at Johns Hopkins	Locally Advanced	30	NCT03767582

Supplementary Table S2. Actively recruiting phase 1 trials incorporating neoadjuvant radiation therapy for pancreatic adenocarcinoma registered on ClinicalTrials.gov.

Study Title	Sponsor	Resectability Status	Estimated Enrollment	ClinicalTrials.gov Identifier
Intraoperative Radiation Therapy After Stereotactic Body Radiation Therapy and Chemotherapy in Treatment of Pancreatic Adenocarcinoma	Sidney Kimmel Comprehensive Cancer Center at Johns Hopkins	Resectable, Borderline Resectable, or Locally Advanced	25	NCT05141513
SHAPER: A Phase 1 Study of Losartan and Hypofractionated Radiation Therapy After Induction Chemotherapy for Borderline Resectable or Locally Advanced Pancreatic Cancer	University of Utah	Borderline Resectable or Locally Advanced	23	NCT04106856
A Phase I Dual Dose Escalation Study of Radiation and Nab-Paclitaxel in Patients With Unresectable and Borderline Resectable Pancreatic Cancer	Abramson Cancer Center at Penn Medicine	Borderline Resectable or Locally Advanced	42	NCT02207465
Phase I Study of Concurrent Nab-Paclitaxel + Gemcitabine With Hypofractionated, Ablative Proton Therapy for Locally Advanced Pancreatic Cancer	University of Maryland, Baltimore	Locally Advanced	24	NCT03652428
Preoperative, Proton-Radiotherapy Combined With Chemotherapy for Borderline Resectable Pancreatic Cancer	EBG MedAustron GmbH	Borderline Resectable	10	NCT04894643

Supplementary Table S3. Active but not-yet-recruiting trials incorporating neoadjuvant radiation therapy for pancreatic adenocarcinoma registered on ClinicalTrials.gov.

**Study Title**	**Sponsor**	**Resectability Status**	**Estimated Enrollment**	**ClinicalTrials.gov Identifier**
Stereotactic Body Radiation Therapy Followed by NALIRIFOX vs NALIRIFOX for Borderline Resectable Pancreatic Cancer	Tianjin Medical University Cancer Institute and Hospital	Borderline Resectable	96	NCT06259058
AG Combined With Immunotherapy and SBRT in Patients With Potentially Resectable Pancreatic Cancer	The Affiliated Nanjing Drum Tower Hospital of Nanjing University Medical School	Potentially Resectable	108	NCT06080854
Phase I+Phase II Clinical Study of PRaG Therapy in Combination With Chemotherapy (AG Regimen) for Neoadjuvant Treatment of Locally Advanced Pancreatic Ductal Adenocarcinoma (PDAC) (NeoPRAG Study)	Second Affiliated Hospital of Soochow University	Borderline Resectable or Locally Advanced	66	NCT06345599
Neoadjuvant Triple Treatment With FOLFIRINOX Plus Pembrolizumab and SABR in Patients With Borderline Resectable Pancreatic Cancer (PREOPANC-5): A Multicenter Single Arm Phase I/II Trial of the Dutch Pancreatic Cancer Group	Amsterdam UMC, location VUmc	Borderline Resectable	66	NCT06384560
A Prospective, Single-arm, Exploratory Phase II Clinical Study Evaluating the Efficacy of Gemcitabine and Nab-palitaxe Combined With Cadonilimab Sequential Short-course Radiotherapy in the Treatment of Patients With Locally Advanced Pancreatic Ductal Adenocarcinoma	Tianjin Medical University Cancer Institute and Hospital	Locally Advanced	30	NCT06472037
